# Supplementary material for: Development of a computer-aided design software for the quantitative evaluation of aesthetic damage
Source: PLoS One. 2019 Dec 18;14(12):e0226322. doi: 10.1371/journal.pone.0226322 (PMC6919621; doi:10.1371/journal.pone.0226322)
Supplement: S1 Code site — 1. (ZIP) [file pone.0226322.s001.zip › [EESC_jr][Projeto]Relatorio_07_21/reports/2/2017.02.16/07:40:08/report.pdf]

Preâmbulo

Na data de 16/02/2017 no horário 09:00, o Perito(a) Professor Doutor Nelson Sakaguti, Cirurgião Dentista, designado(a)para proceder ao exame pericial solicitado em: mazé mané, do gênero Feminino, nascido(a) em 31/10/1957, ocupação do lar, portador(a) do RG 1234567890 sss, cujo endereço rua jaburu, passa a expor a avaliação de dano estético corporal.

Exame objetivo da lesão

A lesão ora objeto de análise teve como natureza Acidente pessoal ocorrido em 20/12/2016. O prejuízo estético consiste em lesão cicatricial consolidada na região: Nariz, Orelhas no lado esquerdo, direito.

Trata-se de cicatriz Hipertrófica de tamanho/frequência Única: Retilínea de orientação Horizontal de pigmentação Normocrômica medindo 1 centímetro(s) na horizontal, 3 centímetros na vertical e 2 milímetros de Profundidade. De contornos Regulares. Perceptivel em 50 centímetros de distância nas relações íntimas. Perceptivel em 3 metros nas relações sociais. A lesão tem repercução na vida diária afetiva, familiar, social conforme fotos anteriores e atuais..

Conclui-se que classificada segundo os parâmetros de dano estético AIPE - Análise do Impacto do Prejuízo Estético (escala de seis níveis e pontuação entre 0 e 50 pontos), como sendo dano estético relativo à área 1, a lesão é caracterizada por 3 pontos e grau de prejuízo estético: Leve.

Escore final: 3;
Grau de prejuízo: Leve.

Faculdade de Odontologia da USP, 16/02/2017

____

Professor Doutor Nelson Sakaguti
